# Developmental Trajectories Leading to Hostility Toward Women: A Structural Equation Modeling Study

**DOI:** 10.1177/10778012241254851

**Published:** 2024-05-24

**Authors:** Caroline Deli, Etienne Garant, Alexandre Gauthier, Jean Proulx

**Affiliations:** School of Criminology, 5622 Université de Montréal, Montréal, Québec, Canada

**Keywords:** hostility toward women, psychological factors, emotional negativity, hostile masculinity, structural equation modeling

## Abstract

Hostility toward women is frequently examined as a risk factor for violence against women, but research on its antecedents is sparse. The aim of this study was to explore the developmental and psychological antecedents associated with hostility toward women in a Canadian sample of sexual aggressors of women. Drawing on Malamuth’s confluence model of sexual aggression, we developed a multifactorial model of hostility toward women, using structural equation modeling. The results indicate the presence of three trajectories, all starting from childhood victimization and leading to hostility toward women, involving antisocial characteristics, emotional negativity, anxiety, and depression.

Check (1988) defines hostility toward women as a hostile attitude which is specifically directed toward women and encompasses beliefs such as “women are deceitful or manipulative.” Such attitudes are often expressed through violent acts targeting women. In fact, hostility toward women is a well-established risk factor for sexual aggression of women (Marshall & Hambley, 1996; Marshall & Moulden, 2001; Murnen et al., 2002; Ray & Parkhill, 2023; Seto et al., 2023), and is also associated with other forms of violence perpetrated by men against women (O’Neil & Harway, 1997; Schippers & Smid, 2021).

## Antisociality and Hostile Masculinity

A large body of literature highlights the importance of hostility toward women in predicting sexual aggression. To explain this relationship, feminist studies investigating the relationship between a broader male ideology (e.g., rape-myth acceptance, hostility toward women) and sexual assault frequently draw on a sociocultural model of rape. This model emphasizes the patriarchal organization of society as a key factor contributing to the perpetuation of sexual violence against women: men are encouraged to be dominant over women, who are compelled to assume more passive and submissive roles (Burt, 1980; Gallagher & Parrott, 2011; Murnen et al., 2002; O’Neil & Harway, 1997). In this view, sexual assault and violence toward women are means of exerting men's dominance over women (Burt, 1980; Gallagher & Parrott, 2011; Proulx & Beauregard, 2014). Central to this body of literature are certain concepts of masculinity—involving, for example, adversarial sexual beliefs (i.e., attitudes toward gender roles)—and rape-myth acceptance.

Some studies have examined whether coercive sexual behaviors against women, such as sexual assault, can be explained in terms of “masculine” traits or characteristics. For example, Murnen et al. (2002) conducted a meta-analysis that identified which personality features related to masculine ideology are most strongly related to sexual aggression. Their results show that Malamuth et al.'s (1991) “hostile masculinity” construct and Mosher's “hypermasculinity” construct (e.g., Mosher & Anderson, 1986) are strongly correlated to sexual aggression. In addition, a recent systematic review by Ray and Parkhill (2023) indicates a broad consensus on the association between “hostile masculinity” and sexual aggression.

Malamuth et al.'s (1991) confluence model of sexual aggression incorporates “hostile masculinity,” which integrates variables related to hostile and distrustful traits, and variables related to the desire to exert dominance over women. Malamuth's model is based on research on hostile masculinity which used a variety of scales, including the Adversarial Sexual Beliefs Scale (Burt, 1980), the Hostility Toward Women Scale (Check, 1988), the Sexual Dominance Scale (Nelson, 1979), and the Negative Masculinity Scale (Spence et al., 1979). While the first three scales measure hostile tendencies specifically directed toward women, the Negative Masculinity Scale is a more general assessment of masculinity and hostile attitudes, and measures personality characteristics such as arrogance, greed, cynicism, and egotism (Spence et al., 1979). According to Malamuth et al. (1993, 1996), hostile masculinity is related not only to psychopathic traits—such as a tendency to manipulate, a lack of empathy, and aggressiveness—but also to some narcissistic traits (mostly due to characteristics measured by the Negative Masculinity Scale). Hostile masculinity is present in one of the two trajectories of the confluence model of sexual aggression. This trajectory starts with childhood victimization, which contributes to the development of delinquency, which in turn contributes to attitudes supporting violence, hostile masculinity, and, ultimately, sexual violence against women (Malamuth et al., 1991, 1996).

Studies based on the confluence model further highlight the relationship between hostility toward women and more general antisocial–psychopathic traits. According to Knight and Sims-Knight (2003), “hostile masculinity” is similar to psychopathy in that both concepts are not homogeneous constructs and both comprise two subcomponents which concord well with the two factors of the PCL-R (Hare & Neumann, 2008). They thus divide hostile masculinity into a component related to insensitivity and lack of empathy on the one hand, and a component related to antisocial behavior on the other. Through structural equation modeling (SEM) and replication of Malamuth's confluence model, Knight and Sims-Knight (2003) demonstrate that this clarification of the concept of hostile masculinity leads to a better fit of the data than does the original model based on the undivided construct of hostile masculinity.

The link between hostile masculinity and psychopathy is further supported by research about sexual victimization that examines variables such as lack of empathy, narcissism, and antisocial behavior (LeBreton et al., 2013; Malamuth et al., 2021; Marshall & Moulden, 2001). When examining predictors of sexual and nonsexual aggression, Hunter et al. (2010) found hostile masculinity to be strongly associated with psychopathic and antagonistic traits. In that study, hostile masculinity was measured by the Hostility Toward Women Scale (Check, 1988), the Adversarial Sexual Beliefs Scale (Burt, 1980), the sexual function index of the Moral Disengagement Scale (Bandura et al., 1996), and the revised Attraction Scale (Malamuth, 1989). Furthermore, LeBreton et al. (2013) found a large array of negative attitudes toward women to be reliably predicted by antisocial and psychopathic personality traits. More specifically, hostility toward women was significantly associated with all the psychopathy-related traits examined, with trait anger and narcissistic entitlement accounting for most of the predicted variance.

## Emotional Negativity

While antisociality is undeniably linked to hostility toward women, other characteristics also seem to play a role in determining this attitude. Thus, several empirical studies highlight a relationship between hostility toward women, on the one hand, and negative emotions (toward oneself and/or others) and adverse life experiences, on the other. Lack of social relationships or intimacy is associated with hostility toward women: feelings of loneliness or isolation are correlated not only with higher levels of hostility in general but also with hostility specifically directed toward women (Check & Malamuth, 1985; Malamuth et al., 1991; Marshall & Hambley, 1996; Seto et al., 2023). Marshall (1989) suggests that the inability to form intimate relationships with women causes men to become emotionally isolated, resulting in feelings of loneliness and frustration. This frustration can be expressed through violent behavior (e.g., aggression, sexual assault) directed at women (Marshall, 1989).

In addition, men who exhibit high levels of hostility toward women often feel inadequate or powerless (Cowan & Mills, 2004). Low self-esteem and lack of personal well-being (Boden et al., 2007; Cowan & Mills, 2004; Mills, 2003), as well as a lack of hope for the future and a sense of incompetence (Williams & Arntfield, 2020) are also associated with hostility toward women. Dissatisfaction with oneself or one's own life, therefore, appears to be associated with this attitude.

## Developmental Factors

Experiences of childhood abuse (e.g., physical, psychological, or sexual victimization as a child) are frequently reported in studies of hostile attitudes or behaviors toward women. A systematic review by Tharp et al. (2013) identifies “childhood maltreatment” as one of the strongest risk factors for adult-female sexual assault. Moreover, this risk factor is frequently included in etiological models of sexual assault (e.g., Hunter et al., 2010; Knight & Sims-Knight, 2003; Malamuth et al., 1996). Malamuth's confluence model of sexual aggression outlines a developmental sequence that includes victimization in childhood and delinquency (Malamuth et al., 1991, 1996). Moreover, Burt (1980) reports childhood victimization to be an antecedent to hostile attitudes toward women, and Vivolo-Kantor et al. (2013) indicate that hostility toward women mediates the relationship between childhood victimization and sexual assault.

## Relevance of the Study

Hostile attitudes toward women are thought to be related not only to antisocial characteristics but also to adverse life experiences and negative feelings about oneself or one's environment. Thus, a set of developmental, cognitive, and affective factors are thought to be associated with this attitude. However, these characteristics have rarely been studied together or integrated into a single model that accounts for their respective influences on behavioral or attitudinal variables. In addition, few studies have examined hostility toward women's antecedents and development (Cowan & Mills, 2004; Gallagher & Parrott, 2011).

## Aim of the Study

We were interested in how a diversity of factors reported in the literature—notably in the confluence model of sexual assault developed by Malamuth et al. (1996) and revised by Knight and Sims-Knight (2003)—are related to hostility toward women. Thus, the overall goal of the study was to develop an explanatory model of hostility toward women in sexual aggressors of women. More specifically, we sought to develop an integrative model that incorporates variables mentioned in the literature on hostility toward women and is capable of shedding light on (a) the role of variables related to hostile masculinity and antisociality; (b) the influence of negative emotional characteristics such as low self-esteem, depression, and anxiety; and (c) the impact of developmental variables such as childhood victimization and delinquency.

## Method

### Data Source

Our sample is composed of 200 participants who received a sentence of 2 years or more for a sexual offense against an adult woman (16 years or older) committed between 1995 and 2000 in Quebec. The sample was recruited from the Regional Reception Centre (Sainte-Anne-des-Plaines, Quebec) and other Quebec penitentiaries (for details on the sample composition, see Proulx et al., 2014). Before participating in the study, all participants gave their consent for the information collected to be used for research purposes. Each participant completed a series of psychometric tests and participated in several semi-structured interviews. Information on participants’ life history, sexuality, family history, work, and criminal career, and on precrime factors and modus operandi, was collected using the Computerized Sex Offender Questionnaire (CSOQ; St-Yves et al., 1994). Official data, such as police reports or victim-impact statements, were used to supplement the information collected in the interviews. In case of discrepancies, official information was retained.

### Participants

The 200 sexual aggressors of adult women (34 sexual murderers and 166 nonhomicidal sexual aggressors) who participated in this study were between the ages of 18 and 68 years (*M *= 33.49, *SD *= 9.06), and predominantly French-speaking (88.5%), White (86%), and single (68.5% separated, divorced, single, or widowed; 31.5% married or with a nonmarital intimate partner). Hostility toward women 48 hours and/or 1 year before the index offense was reported by 58% (*n *= 116) of the sample.

### Measures

#### Hostility Toward Women

Hostility toward women was assessed by an experienced psychologist who used CSOQ criteria to determine whether respondents had experienced marked unfavorable feelings toward, or conflict with, women 48 hours or 1 year prior to committing their index offense. The dependent variable, “hostility toward women,” was dichotomous and was coded “*yes*” (1) when the participant reported such conflict and “*no*” (0) otherwise. In the CSOQ, “Conflict” is defined as a state of tension, disagreement, or internal struggle expressed in behaviors of opposition, confrontation, or avoidance. In this study, these behaviors had to have been directed toward women in general, rather than specifically at the victim of the offense.

#### Developmental and Adulthood Variables

Several variables concerning events and behaviors present in childhood and adolescence (before age 18) were used. All variables were dichotomous, taking the value of 1 when the *event had occurred* and 0 when *it had not*. Some variables were related to childhood: exposure to physical victimization, exposure to emotional victimization, exposure to sexual victimization, actual physical victimization, actual emotional victimization, and actual sexual victimization. Other variables concerned behaviors in both childhood and adolescence: temper tantrums, reckless behavior, and rebellious attitude. In addition, several variables pertaining to situations experienced in adulthood were used: low physical self-esteem, low psychological self-esteem, and low self-esteem. All variables were dichotomous and took the value of 1 when the *situation was present* and 0 when *it was absent*.

#### Psychometric Instruments

Participants completed several psychometric instruments whose scores are reported to be associated with hostility toward women. The Minnesota Multiphasic Personality Inventory (MMPI-2; Butcher et al., 1989) consists of 566 questions and comprises 10 psychopathology-related clinical scales: hypochondria (anxiety related to bodily symptoms), depression (depressive affect), hysteria (physical problems related to psychological vulnerabilities), psychopathic deviate (lack of empathy and impulsivity), masculinity/femininity (traditional gender attitudes), paranoia (excessive distrust), psychasthenia (anxiety, ruminations, and tension), schizophrenia (alienation, unusual behaviors or thoughts), hypomania (mental and physical excitability), and social introversion (social withdrawal). The mean for each scale is set at 50 and a T-score of 70 (two standard deviations above the mean of the population) is the threshold for significant psychopathology.

The Carlson Psychological Survey (CPS; Carlson, 1981) is a psychological assessment questionnaire developed for prison populations with behavioral or substance-abuse problems. Participants answer 50 items using a 5-point Likert scale and are evaluated in terms of the following four dimensions: substance abuse (degree of drug or alcohol abuse), thought disturbance (perceptual distortions, confusion, anxiety), the antisocial tendency (hostility, threatening behavior), and self-deprecation (poor self-esteem).

The Beck Depression Inventory (BDI; Beck et al., 1987) evaluates the severity of the depression of respondents, who indicate the extent to which different symptoms are present in their lives, on a 4-point Likert scale. The BDI has 21 items. The higher the score, the more severe the depressive symptoms (Beck et al., 1987).

Finally, the State-Trait Anxiety Inventory (STAI; Spielberger et al., 1971) is a questionnaire composed of 20 questions measuring state anxiety (i.e., the state of tension, nervousness and worry felt at a given moment) and 20 questions measuring trait anxiety (i.e., the general tendency to anxiety). For each question, participants indicate on a 4-point Likert scale the extent to which the question applies to them. The higher the score, the higher the anxiety (Spielberger et al., 1971).

### Analyses

Initially, descriptive and bivariate statistics were performed with IBM SPSS Statistics Version 27. For all the instruments used, participants who reported hostility toward women were compared with participants who did not report this attitude. The significance of observed differences was analyzed using chi-square tests and Student's *t*-tests. The phi (φ) coefficient and eta squared (η^2^) were used as indicators of the effect size for the chi-square tests and the Student's *t*-tests respectively. The Mann–Whitney U-test was used when the assumptions of parametric statistics were not satisfied (Cohen, 2013). This was the case when data were not normally distributed (according to indicators of skewness and kurtosis), or when Levene's test indicated that the assumption of homogeneity of variance was not satisfied.

A second set of analyses was performed using Mplus Version 8.4. Since the rate of missing data varied from 0% to 45.5%, we began by performing multiple imputations with 20 iterations before conducting the rest of our analyses. Multiple imputation is a statistical technique used to manage missing data. Using the data available, it predicts a value for each missing data item (Allison, 2003; Cox et al., 2014). This technique is particularly useful when performing SEM (Allison, 2003), and is not expected to affect the quality of the results for a sample size of 200, even when the missing data rate is 40% (Enders & Mansolf, 2018).

SEM was then performed. This statistical modeling technique relies on factor analyses and regressions to identify complex trajectories that combine latent factors and manifest variables (Hox & Bechger, 1998; Longpré et al., 2018). The factor structure of our model of hostility toward women was tested using confirmatory factor analyses (CFAs).

**Model Fit**. Three fit indices were used to test the models of hostility toward women: the root mean square error of approximation (RMSEA), the comparative fit index (CFI), and the Tucker–Lewis index (TLI). The RMSEA is an absolute fit index that compares the model being tested to the best possible model, for which the fit index would be 0 (Kline, 2015). The CFI and TLI are relative fit indices that measure how well the tested model fits, by comparing it to a baseline model in which none of the variables are correlated with each other (Hu & Bentler, 1999; Kline, 2015). The lower the RMSEA and the higher the CFI and TLI, the better the model tested is. In general, a RMSEA between .05 and .08 is considered acceptable, and a value below .05 is considered excellent (Wang & Wang, 2020). A value between .90 and .95 for CFI and TLI indicates a well-fitting model (Hu & Bentler, 1999; Wang & Wang, 2020).

**Model Modification**. The model was constructed and refined using the fit indices provided by Mplus. Initial models included a latent variable of exposure to pornography (movies or magazines) during childhood, and manifest variables of actual, and exposure to, sexual victimization during childhood. These models were discarded due to low fit scores. The latent variable of exposure to pornography and the manifest variables pertaining to sexual victimization were also discarded, as the factor loadings were unsatisfactory. Various solutions to the measurement model were tested by adding or removing variables, on the basis of model-fit indices provided by Mplus. Only suggestions with theoretical relevance were retained. Following the same procedure, based on fit indices and theoretical relevance, we tested several solutions for the structural model.

## Results

### Univariate and Bivariate Analyses

Univariate and bivariate statistics are reported in Table 1. Percentages are reported for the total sample, for individuals hostile toward women and individuals not hostile toward women. Variables reported include developmental variables (for childhood, adolescence, and adulthood), and results on psychometric instruments.

**Table 1.** Descriptive and Bivariate Statistics (*N* = 200).

Variables	Hostility Toward Women (%)					
	Yes (*n* = 116)	No (*n* = 84)	χ^2^	*p*	φ	
**Childhood victimization**						
Exposure to psychological victimization	63.1	45.7	5.93	*	.17	
Exposure to physical victimization	64.3	37.1	14.45	***	.27	
Exposure to sexual victimization	19.0	5.2	9.58	**	.22	
Psychological victimization	69.0	43.1	13.20	***	.26	
Physical victimization	63.1	42.2	8.48	**	.21	
Sexual victimization	50.0	24.1	14.32	***	.27	
**Behavioral problems (childhood and adolescence)**						
Temper tantrums	43.9	31.9	2.96	*ns*	.12	
Reckless behavior	26.8	18.4	1.97	*ns*	.10	
Rebellious attitude	60.2	49.1	2.39	*ns*	.11	
**Adulthood variables**						
Low self-esteem	59.8	37.2	9.75	**	.22	
Low physical self-esteem	22.6	13.8	2.63	*ns*	.12	
Low psychological self-esteem	60.7	36.2	11.76	***	.24	

*Note.* ns = not significant.

* *p < *.05. ** *p < *.01. *** *p <* .001.

### Victimization During Childhood

In childhood, individuals reporting hostility toward women were significantly more likely to be exposed to psychological (χ^2 ^= 5.93, *p *< .05), physical (χ^2 ^= 14.45, *p *< .001), and sexual (χ^2 ^= 9.58, *p *< .01) victimization than were individuals not reporting this attitude (Table 1). In addition, they were more likely to experience psychological (χ^2 ^= 13.20, *p *< .001), physical (χ^2 ^= 8.48, *p *< .01), and sexual (χ^2 ^= 14.32, *p *< .001) victimization. The difference in the effect size of these two groups ranged from small (φ = .17 for exposure to psychological victimization) to moderate (φ = .27 for exposure to physical victimization; φ = .26 for psychological victimization; φ = .21 for physical victimization).

### Behavioral Problems in Childhood and Adolescence

The prevalence of variables related to behavioral problems in childhood and adolescence did not significantly differ in individuals who reported hostility toward women and individuals who did not report this attitude (Table 1).

#### Self-esteem in Adulthood

Men who reported hostility toward women were significantly more likely to have low self-esteem in adulthood than those who did not (59.8% vs. 37.2%; χ^2 ^= 9.75, *p *< .01; Table 1). The effect size for this difference was moderate (φ = .22). The same pattern was observed for low psychological self-esteem (hostility: 60.7% vs. no hostility: 36.2%; χ^2 ^= 11.76, *p *< .001). Again, the effect size for this difference was moderate (φ = .24). There was no significant difference between these two groups for low physical self-esteem.

#### Psychometric Instruments

Only 139 participants completed the MMPI-2 (Table 2). Several significant differences were found between the MMPI-2 scores of men who reported hostility toward women and those who did not. Men who reported hostility toward women were significantly more likely to score high on the hypochondria (χ^2 ^= 8.96, *p *< .01), depression (χ^2 ^= 10.61, *p *< .001), psychopathic deviate (χ^2 ^= 5.17, *p *< .05), psychasthenia (χ^2 ^= 7.87, *p *< .01), and schizophrenia (χ^2 ^= 8.98, *p *< .01) scales (Table 2). For all these differences, the effect size was moderate (φ between .19 and .28). There were no significant differences between these two groups on the hysteria, masculinity/femininity, paranoia, hypomania, and social introversion scales.

**Table 2.** Distribution of MMPI-2 Scores (*N* = 139).

	Hostility Toward Women (%)				
	Yes (%) (*n *= 55)	No (%) (*n *= 84)	χ^2^	*p*	φ
Score > 70					
Hypochondria	32.7	11.9	8.96	**	.25
Depression	40.0	15.5	10.61	***	.28
Hysteria	23.6	14.3	1.97	*ns*	.12
Psychopathic deviate	52.7	33.3	5.17	*	.19
Masculinity/femininity	1.8	1.2	0.09	*ns*	.03
Paranoia	52.7	42.9	1.30	*ns*	.10
Psychasthenia	34.5	14.3	7.87	**	.24
Schizophrenia	45.5	21.4	8.98	**	.25
Hypomania	16.4	11.9	0.56	*ns*	.06
Social introversion	12.7	8.3	0.71	*ns*	.07

*Note.* MMPI = Minnesota Multiphasic Personality Inventory; ns = not significant.

* *p* < .05. ** *p* < .01. *** *p* < .001.

Men who reported hostility toward women had significantly higher mean scores on the CPS thought disruption scale (T = −2.26, *p *< .05) and self-deprecation scale (T = −2.07, *p *< .05) than did men who did not report hostility (Table 3). Similarly, men who reported hostility toward women had significantly higher median scores on the Beck Depression Inventory than did men who did not (U = 1730.5, *p *< .05; Table 4). However, the two groups did not have significantly different scores on the STAI trait anxiety and situational anxiety scales (Table 3).

**Table 3.** Distribution of Carlson Psychological Survey and STAI Scores (*N* = 200).

	Hostility Toward Women						
	Yes (*n *= 84)		No (*n *= 116)				
Variables	*M*	*SD*	*M*	*SD*	T	*p*	η^2^
**Carlson Psychological Survey**							
Substance abuse	21.18	6.66	19.70	6.82	−1.27	*ns*	.23
Thought disturbance	33.21	8.43	30.02	8.03	−2.26	*	.30
Antisocial tendency	29.73	5.36	28.14	5.39	−1.71	*ns*	.18
Self-deprecation	19.86	5.60	18.04	4.72	−2.07	*	.17
**STAI**							
Trait anxiety	46.62	14.28	42.04	12.90	−1.91	*ns*	.34
State anxiety	50.04	15.03	46.12	15.33	−1.46	*ns*	.42

*Note.* STAI = State-Trait Anxiety Inventory; ns = not significant.

* *p* < .05. ** *p* < .01. *** *p* < .001.

**Table 4.** Beck Depression Inventory Scores (*N* = 200).

Hostility Toward Women						
Yes (*n *= 84)		No (*n *= 116)			** **	
*M* (*SD*)	*Mdn* (IQR)	*M* (*SD*)	*Mdn* (IQR)	U (z)	*p*	*r*
19.51 (12.54)	18.50 (19)	14.89 (10.78)	13 (14)	1,730.5 Z = −2.00	*	−.17

*Note.* ns = not significant.

Mann Whitney's U-test was performed, due to nonhomogeneity of variance.

* *p < *.05. ** *p < *.01. *** *p < *.001.

### Structural Equation Modeling

#### Measurement Model

The measurement model was developed using SEM, and CFA was later used to assess the validity of the measurement model. Our measurement model consisted of five latent variables, each of which was measured by three to four manifest variables. These variables were chosen based on their theoretical relevance to each latent variable and the goodness of fit indicated by Mplus. The strength of the relationship between a latent variable (factor) and the manifest variables (indicators) is indicated by factor loadings (i.e., standardized regression coefficients). The higher the factor loading, the more an indicator contributes to the factor. The first latent variable, “victimization during childhood” was based on four manifest variables: exposure to physical victimization, exposure to psychological victimization, physical victimization, and psychological victimization. The factor loadings were all significant (*p *< .001) and ranged from .918 to .980. The latent variable “behavior problems” was based on behavioral problems during childhood and adolescence. This latent variable comprised three manifest variables: temper tantrums, reckless behavior, and rebellious attitude. The factor loadings were all significant (*p *< .001) and ranged from .542 to .891. The latent variable “emotional negativity” was based on low physical self-esteem, low psychological self-esteem, and low self-esteem in adulthood. The factor loadings were all significant (*p *< .001) and ranged from .772 to .946. The latent variable “antisocial characteristics in adulthood” was based on scores on two scales of the MMPI-2—the psychopathic deviate scale and the schizophrenia scale—as well as the CPS’ antisocial tendencies scale. These three manifest variables had significant (*p *< .001) factor loadings ranging from .409 to .736. Finally, our model included the latent variable “anxiety and depression” in adulthood. Three manifest variables loaded with this factor: the trait anxiety scale of the STAI, the depression scale of the MMPI-2, and the Beck Depression Inventory score. All factor loadings were significant (*p *< .001) and ranged from .664 to .737.

The results of confirmatory factor analyses indicated that the measurement model had an acceptable fit to the data; the CFI and TLI indicated a good fit (CFI = .929, TLI = .912), although the RMSEA did not reach an acceptable value (RMSEA = .097).

#### Structural Model

**Figure 1.**
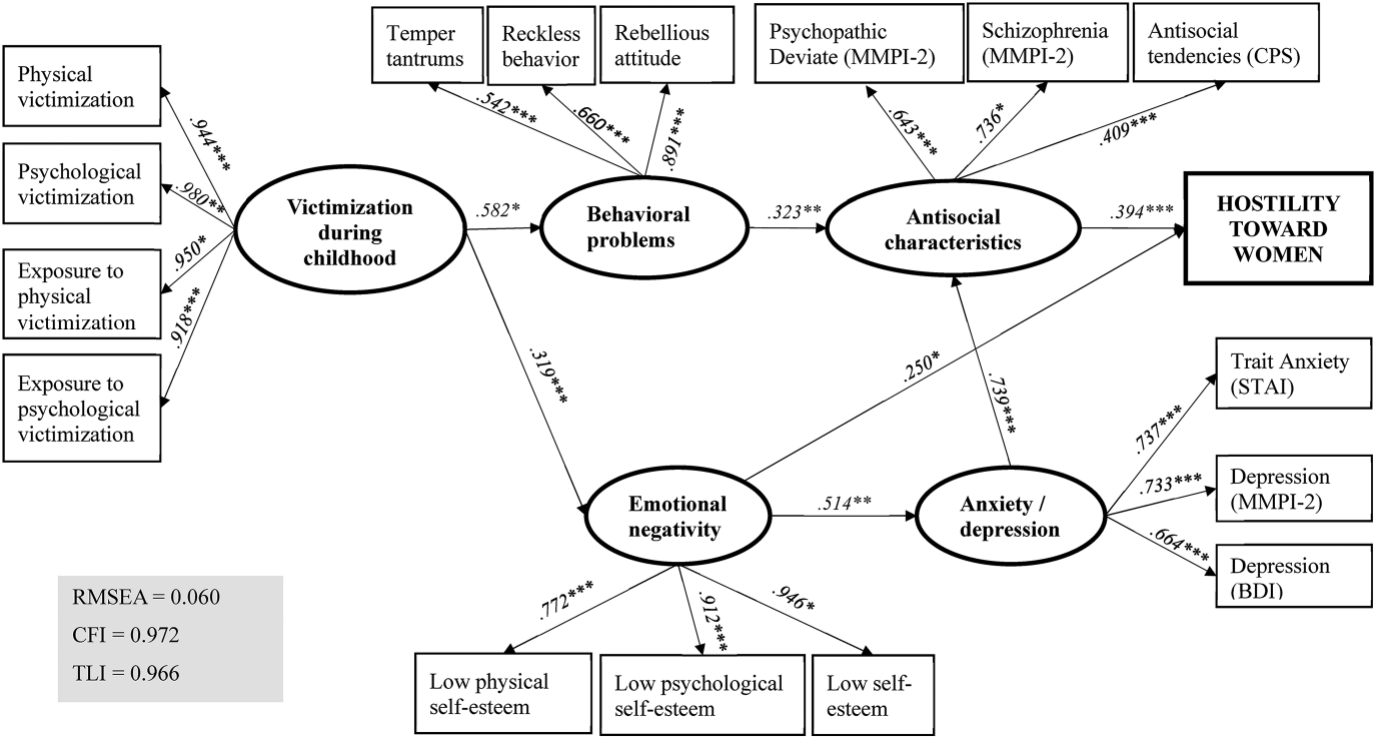
Structural equation model.

*Note.* * *p* < .05. ** *p* < .01. *** *p* < .001.

Figure 1 presents the results of our SEM. The fit indices are excellent and indicate that our model fits the data (RMSEA = .060, CFI = .972, TLI = .966). This model suggests the presence of three trajectories leading to hostility toward women. The first trajectory indicates that the latent variable “victimization during childhood” leads to the latent variable “behavioral problems” (β = .582, *p *< .001), which subsequently leads to the development of antisocial and hostile characteristics in adulthood (β = .323, *p *< .001) and, ultimately, hostility toward women (β = .394, *p *< .001). The second trajectory begins with “victimization during childhood,” which leads to “emotional negativity” (β = .319, *p *< .001) in adulthood and, ultimately, hostility toward women (β = .250, *p *< .05). Finally, the third trajectory also begins with “victimization during childhood,” which subsequently leads to “emotional negativity” in adulthood (β = .319, *p *< .001), “anxiety and depression” in adulthood (β = .514, *p *< .001), “antisocial characteristics” in adulthood (β = .739, *p *< .001), and, finally, hostility toward women (β = .394, *p *< .001).

## Discussion

Characteristics that shed light on the development of hostility toward women have emerged from the literature. These include, on the one hand, variables related to antisociality, psychopathy, and general hostility and, on the other, variables related to negative emotions toward oneself or others. In terms of developmental factors, variables related to the presence of victimization during childhood and to delinquency have also emerged in the literature. Based on this body of research, the objective of our study was to develop an integrative model of hostility toward women that incorporates these factors.

Our results revealed significant differences between individuals who reported hostility toward women and those who did not. Individuals who reported hostility toward women appear to have experienced different forms of victimization during childhood. In adulthood, they are distinguished by low self-esteem, particularly in terms of their personality. Their scores on psychometric instruments indicate that they are more preoccupied and anxious, have a greater tendency to depression, lack empathy, and are more impulsive and aggressive.

Our SEM highlights the presence of three trajectories that lead to hostility toward women. The first trajectory, which includes the developmental variables of “victimization during childhood” and “behavioral problems,” appears to have elements similar to Malamuth et al.'s (1996) trajectory of “hostile masculinity.” Whereas Knight and Sims-Knight's (2003) model includes an antisocial-behavior component and an emotional-detachment component, it is antisociality that is predominant in our trajectory and is particularly associated with aggressive and impulsive characteristics, as can be seen in the high scores on the psychopathic deviate and schizophrenia scales of the MMPI-2. Interestingly, a personality profile characterized by high scores on these two scales has been reported to be associated with not only violent sexual aggression but also superficial emotions and callousness (Moseman, 2014). Consequently, our results appear to be consistent with Knight and Sims-Knight's model.

The second trajectory begins with “victimization during childhood” and indicates a direct link between emotional negativity and hostility toward women. Variables related to low self-esteem are frequently studied in relation to interpersonal violence. Studies by Marshall (e.g., Marshall & Moulden, 2001) highlight a link between sexual assault and low self-esteem. Specifically, low self-esteem is thought to limit the ability to develop adequate interpersonal relationships and intimate bonds, and thus to favor aggressive attitudes that lead to coercive behaviors toward women (Marshall & Moulden, 2001). In a prospective longitudinal study, Díaz-Faes and Widom (2024) indicate that children who experienced maltreatment were more likely to perpetrate physical intimate partner violence later in life and that this relationship was mediated by low self-esteem. They suggest that, because people with low self-esteem were less assertive and less secure in their relationships, violently acting out was a way to regain control over their relationships. Although several studies report a positive relationship between low self-esteem and violent behavior, the strength of this relationship may be reduced when control variables related to family and psychosocial context are taken into account (Boden et al., 2007). Our results do not explain the relationship between self-esteem and violent behavior, but they do indicate that lower self-esteem is associated with the development of hostile attitudes toward women.

Finally, our third trajectory involves “anxiety and depression” in adulthood, which is linked to antisociality, and, ultimately, hostility toward women. This trajectory has elements very similar to one of the trajectories identified by Hunter et al. (2010), who point out that psychosocial deficits such as low self-esteem, anxiety, and depression lead to antisocial characteristics and, subsequently, hostile masculinity.

Our results confirm the relationships found in the literature between hostility toward women, more general antisociality, and negative feelings toward oneself and others. Furthermore, our results integrate these different factors into a model which clarifies the relationships found in the literature. Physical and psychological victimization in childhood and behavioral problems in adolescence are also associated with the development of hostility toward women.

### Limitations

There are some limitations to our study that must be considered in the generalization of our findings. First, we specified our theoretical model based on the literature on not only sexual aggression of adult women, but also other types of violent aggression against women. However, our sample consists only of sexual aggressors of women, and this sample may not adequately reflect the full range of characteristics that the literature reports. This sample characteristic also limits us in generalizing our results to other populations.

Some considerations hinder the comparison of our results with those of other studies. Our measure of hostility toward women is a summary assessment by an experienced psychologist who applied CSOQ criteria to the content of extensive interviews with offenders. This type of assessment yields a rich and complex understanding of respondents’ hostility toward women in day-to-day life but does not result in a standardized measure. In contrast, hostility toward women has typically been examined using self-report instruments (e.g., Check, 1988) which although standardized are easily falsified by respondents. For these reasons, comparison with the literature must inevitably be an imperfect exercise.

Additionally, certain variables frequently reported to be pertinent to hostility toward women could not be taken into account in our model, due to the nature of the data to which we had access. This is the case, most notably, for lack of empathy, the importance of which is emphasized by Marshall and Moulden (2001), and feelings of powerlessness, mentioned by Cowan and Mills (2004).

Finally, although the confirmatory factor analyses revealed satisfactory TLI and CFI, it also revealed an unsatisfactory RMSEA. This result may indicate some limitations to the validity of the factor structure (measurement model). In other words, these results could mean that the latent factors present in our SEM model may not be optimal and that some manifest variables may not perfectly represent the latent construct with which they are associated. However, this limitation does not undermine the validity of our SEM model, which does show a good fit otherwise. CFAs give indications on the factorial structure of a model, but they do not account for the relationships between latent variables, which are analyzed using SEM. Thus, the low RMSEA obtained with CFA could be due to the fact that the complexity of the relationships between the latent variables was not sufficiently taken into account.

### Future Studies and Implications

Since empirical support for the association between hostility toward women and sexual aggression has been reported for various populations (Ray & Parkhill, 2023), replicating the results with a different sample would allow for better generalization of the results. Furthermore, expanding the current model by integrating other variables mentioned in the literature could better reflect the complexity of the reality of hostility toward women. Expanding the model by looking at how hostility toward women can predict violence would also be a way to better understand the relationships between attitudes and violent behavior.

Our results suggest that not only anger and hostility are associated with antisociality but that other negative emotions such as depression, anxiety, and low self-esteem must also be considered as precursors and treatment targets in services offered to men who are violent against women. Specifically, what our results highlight is the necessity to consider those variables both separately and simultaneously. The first trajectory is in line with mainstream interventions focusing on a general factor of antisociality to prevent sexual aggression against women (Malamuth et al., 2021), while the second and third trajectories put forward the need to consider self-esteem, anxiety, and depression in prevention approaches. In regard specifically to the second trajectory, investigating how negative emotions directly predict hostility toward women might be of interest to both research and practice. Indeed, while our results and those of other studies (Díaz-Faes & Widom, 2024; Marshall & Moulden, 2001) indicate that these variables are relevant in developing hostility toward women, it is unclear how this operates, and perhaps if other cognitive or emotional factors mediate this relationship. The third trajectory draws attention to the simultaneous presence of variables related to antisocial characteristics, low self-esteem, anxiety, and depression. The cooccurrence of these variables in a single trajectory emphasizes the need to consider interventions focused on behavioral, attitudinal, and emotional factors related to these variables and their interactions. Since our model does not target one violent behavior, but an attitude that underlies several types of violence, these results could contribute to the prevention and study of domestic violence and misogynistic violence. Consequently, attitude change strategies developed in social psychology (Albarracin & Shavitt, 2018) may be a source of inspiration for prevention programs that target attitudes favoring violent behavior against women.
